# Influence of Vehicle Speed on the Characteristics of Driver’s Eye Movement at a Highway Tunnel Entrance during Day and Night Conditions: A Pilot Study

**DOI:** 10.3390/ijerph15040656

**Published:** 2018-04-02

**Authors:** Li Qin, Li-Li Dong, Wen-Hai Xu, Li-Dong Zhang, Arturo S. Leon

**Affiliations:** 1 School of Information Science and Technology, Dalian Maritime University, Dalian 116026, China; ql_qinli@dlmu.edu.cn (L.Q.); xuwenhai@dlmu.edu.cn (W.-H.X.); 2 Department of Civil and Environmental Engineering, University of Houston, Houston, TX 77204, USA; aleon3@Central.UH.EDU; 3 High-Grade Highway Construction Authority of Jilin Province, Changchun 130012, China; zld7296@126.com

**Keywords:** driving safety, eye movement, fixation duration, pupil area, vehicle speed

## Abstract

The aim of this study was to investigate how vehicle speed influences the characteristics of driver’s eye movement at highway tunnel entrances during day and night. In this study, six drivers’ eye movement data (from 200 m before tunnel entrance to 200 m inside tunnel entrance) under five predetermined vehicle speeds (40, 50, 60, 70 and 80 km/h) in the daytime and three predetermined vehicle speeds (40, 60 and 80 km/h) in the nighttime were recorded using the non-intrusive Dikablis Professional eye-tracking system. Pupil size, the average fixation duration time and the average number of fixation were analyzed and then the influence of the vehicle speed on these parameters was evaluated by means of IBM SPSS Statistics 20.0. The results for pupil size in daytime increased when approaching the tunnel entrance, while as for nighttime, pupil size decreased when approaching the tunnel entrance and then increased after entering the tunnel. The pupil size in daytime has a significant negative correlation with vehicle speed, while the pupil size in nighttime did not show a significant association with vehicle speed. Furthermore, the average fixation duration in daytime increased when entering the tunnel, and had a significant negative correlation with vehicle speed. Also, the average number of fixations in daytime decreased when entering the tunnel and has a significant negative correlation with vehicle speed. However, the average fixation duration and the average number of fixations in nighttime did not show any significant association with vehicle speed. Moreover, limitations and future directions of the study are discussed for the further investigation.

## 1. Introduction

Underground road tunnels have been undergoing rapid development to relieve the pressure on ground transportation and to create new road networks [1]. By the end of 2016, China had 15,181 highway tunnels with a combined length of 14.0397 million meters. Among these tunnels, 815 are extra-long (tunnel length greater than 3 km) with a combined length of 3.6227 million meters and 3520 are long tunnels (tunnel length between 1 km and 3 km) with a combined length of 6.0455 million meters [2,3]. 

A highway tunnel is a semi-enclosed space because of its special tubular structure [4,5]. This characteristic of a tunnel leads to a changing process of “bright-dark-bright” when drivers enter the tunnel from the external environment during daytime and vision adaptation lagging phenomenon after entering the tunnel [6,7,8]. This changing process and associated phenomenon directly affect driving performance and driving workload, which in turn may cause traffic accidents [9,10]. Although some research shows that fewer accidents occur in tunnels than on open roads [11,12,13,14,15,16], in general, traffic accidents in a tunnel are of greater severity in terms of injuries and deaths compared to open roads [17,18,19]. Abundant studies show that human factor is one of the most important aspects of road traffic accidents [20,21,22,23,24,25,26]. Studies also show that more than 80% of the information drivers obtain while on the road is through visual perception (95% of which is dynamic information), and hence, drivers’ dynamic visual characteristics are most closely related to traffic accidents [27,28,29,30,31,32]. Moreover, the abundant research found that the accident rates in entrance and exit zones were higher than in the mid-zone of the tunnel [33,34]. In particular, it was found that road traffic accidents are more likely to occur when entering the tunnel than exiting [35,36,37]. And the vehicle speed has a great impact on the traffic accidents [38,39,40]. Thus, study and analysis of drivers’ eye movement characteristics around the tunnel entrance and under different vehicle speed conditions have great significance to safe driving and strategies to reduce traffic accidents.

## 2. Literature Review on Drivers’ Eye Movement Characteristics While Driving

With the importance of visual information for driving and its causal connection with accidents, abundant studies have been conducted on eye movement characteristics of drivers which is related to driving safety [41,42,43,44]. Many of them examined differences between novices and experienced drivers by analyzing drivers’ eye movement parameters [45,46,47]. Some results confirmed that the novice drivers tended to search a small area of the visual scene, and fixated closer to the vehicle [48,49]. Many focused on the effects of road conditions on drivers’ eye movement. Gramann et al. [50] designed an experiment through computer simulated tunnels in a dimly lit room to evaluate variation of the eye movements of drivers in straight segments and when performing turning maneuvers. The results showed that the number of saccades had a positive correlation with the turning angle in the tunnel, and the increase in saccades was stronger during the turns compared to straight segments. In addition, fewer fixations occurred in the turn in comparison to segments before and after the turn. Miyoshi and Nakayasu [51] conducted two experiments with different driving configurations using automobile driving simulators. The first experiment involved a simulated road located in an open and wide field without any buildings and moving objects under clear and sunny conditions. The second experiment involved a simulated road located in an urban environment with buildings, shops, and traffic equipment on the sides of the road under prediction event of a hazard on a sunshine day. In both experiments, the road included straight sections, curves, and turns. The experimental results showed that the mean fixation duration was shortest in the turn event and longest in the straight event. In addition, the frequency of saccade was highest in the turn event and lowest in the straight event.

Besides, studies about drivers’ eye movement behaviors while entering the tunnel can be divided into two main categories as discussed below. The first category groups those studies conducted in a driving simulator or other simulation environments. Akamatsu et al. [52] conducted experiments in real road and driving simulator environments to investigate effects of tunnels and other road structures on driving behavior. These experiments involved ten male subjects that drove the target tunnel and road sections twice in one-way trips at a speed of 60 km/h in daytime and nighttime conditions. Drivers’ behavioral data showed that vehicle acceleration decreased when entering and exiting the tunnel. Interestingly, in simulator experiments, vehicle acceleration decreased only when the vehicle exited the tunnel. Calvi and D’Amico [53] used a driving simulator to virtually replicate eight existing tunnels and recorded various driving parameters of twenty-five drivers to investigate the effects of road tunnels on driving behavior and road safety. The findings showed that the driver pays more attention when driving inside a tunnel, and the crash rate inside road tunnels is definitely lower than outside. Xiao et al. [54] conducted experiments using a driving simulator and an eye tracker device to investigate the relationship between weather conditions and road safety. The results indicated that visibility significantly affected the drivers’ speed control and heavy fog hampered drivers’ ability of traffic signals recognition, which would lead to a higher number of traffic accidents. Although experiments conducted in a driving simulator are cheaper, easy to accomplish and involve lower risk compared to experiments in actual roads [55], they have limitations as drivers’ behaviors can be different than those on actual roads [53].

The second category groups those studies carried out in real road environments. Verwey. WB [56] discussed the effect of tunnel entrance on drivers’ physiological and operation behavior by measuring eye-blinks, heart rate and galvanic skin response. This study found that eye blink rate decreased when entering the tunnel entrance, however, the rate varied for different tunnels. Pan et al. [57] analyzed the drivers’ pupil diameter and moving fixation using the EMR-8B system during daytime. The experimental results of this study indicated that pupil diameter increased rapidly after entering the tunnel entrance. In addition, it was found that the index of moving fixation 50 m before tunnel entrance is much lower than inside of the tunnel. Zhao et al. [58] used nine drivers to study visual information perception and variation during daytime and simulated visual feature variation by BP Neural Network. The experimental data of this study showed that fixation duration and saccade amplitude decreased gradually before tunnel entrance and increased first after entering the entrance and started to change smoothly 250 m inside the tunnel. In contrast, the number of fixation increased when approaching the tunnel entrance, and decreased first and changed slowly after entering the entrance. Yan Ying et al. [30] studied drivers’ fixation variation at the tunnel entrance and inside tunnel sections by analyzing nine drivers’ eye movement data. The results showed that the average fixation duration increased significantly before entering the tunnel, and reduced gradually after entering the tunnel. However, 71.4% drivers’ average fixation number decreased gradually, especially at 100 m before the tunnel entrance. Hu et al. [59] carried out experiments to investigate drivers’ pupil size as driving into a tunnel. Pupil size data of eight male drivers were recorded by an eye tracker device while entering into four different tunnels. Experimental results indicated that drivers’ pupil size increased while the vehicle entered the tunnel, however, significant individual differences existed on drivers’ pupil size. Several studies attained similar findings to the increase of pupil size observed here [60,61,62,63]. Wang et al. [64] investigated the visual characteristics of 18 drivers while entering actual tunnel sections in China. The results indicated that the number of fixations increased rapidly before tunnel entrance, then decreased slightly inside the tunnel and finally increased to a constant value. On the contrary, the average duration of fixation decreased before tunnel entrance, and then increased slightly after entering the tunnel and finally decreased to a constant value. He et al. [65] investigated the influence of different tunnel lighting environments on driving safety by analyzing eye movement parameters. The drivers’ vision area in this study was divided into safe and unsafe areas. The results of this study indicated that the proportion of fixation times and the proportion of total fixation time of safe area decreased with an increase of luminance. Also, the pupil diameter decreased when visual environment luminance increased. Shao et al. [66] conducted a field experiment in Xi’an men tunnel to evaluate the safety of tunnel entrance and exit by measuring drivers’ behaviors. Experimental results showed that pupillary diameter and heart rate changed significantly when black hole (entrance zone) and white hole (exit zone) emerged in the visual field of drivers, which has traffic safety implications. 

Thus far, previous studies focused generally on investigating drivers’ eye characteristics while vehicle entered and exited a tunnel during daytime conditions. Also, there are few experimental studies carried out in nighttime conditions, which are relevant for traffic safety considering that many studies showed that nighttime driving is less safe than daytime driving [67,68,69,70]. To our best knowledge, no studies have been conducted to examine the effect of vehicle speeds on drivers’ eye movement characteristic while entering a tunnel in daytime and nighttime conditions. The main objective of this study is to investigate how drivers’ eye movements are related to different vehicle speeds and whether these relationships are the same during daytime and nighttime conditions. Some suggestions to improve highway safety are also provided based on the research findings. The experiments were conducted in an actual tunnel road located in Jilin Province, China. Six experienced drivers traversed a section from 200 m before the tunnel entrance to 200 m inside the tunnel entrance at different speeds during daytime and nighttime. Drivers’ eye movements were measured using a head-mounted eye tracker device, Dikablis-professional version. The eye movement measures included pupil area, fixation duration, and number of fixations.

## 3. Experimental Methodology

### 3.1. Driving Environment

**Figure 1:**
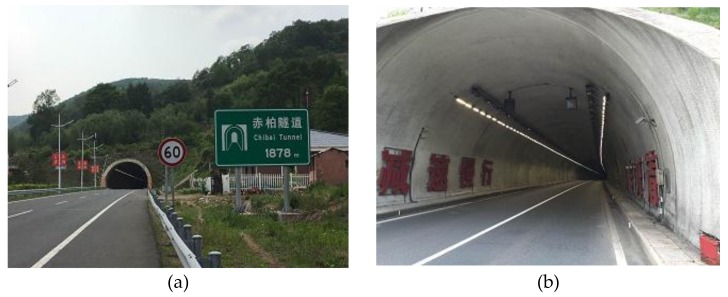
Chibai Tunnel, (**a**) Entrance to the tunnel; (**b**) LED luminaries installed in the tunnel.

The Chibai Tunnel, a section of the Tonghua-Shenyang highway in Tonghua of Jilin Province, China, was chosen as the case study. The tunnel, which is equipped with Light-Emitting Diode (LED) luminaries (Figure 1), consists of two lanes with a length of 1878 m, a width of 10.5 m, and a height of 7.45 m. The design vehicle speed of the tunnel is 80 km/h.

### 3.2. Participants

The driving experiments in the test section involved a greater risk due to its mountainous surroundings, due to the presence of several nearby tunnels, and due to the fact that the road was open to traffic. In order to ensure the driving safety, six experienced male drivers were recruited for this experiment. The six participants are local drivers that often use the tunnel road and hence, these participants may be representative of drivers passing through the tunnel. The basic information of participants is presented in Table 1.

The average age of the participants in Table 1 is 39.5 years, with a standard deviation of 9.4 years. All participants have a valid class “A” driver’s license (driving licenses A, B and C indicate that the holders are allowed to drive a heavy passenger vehicle, heavy goods vehicle, and light motor vehicle, respectively) and have more than 10 years of driving experience. The average driving experience of the participants is 16.5 years with a standard deviation of 7.7 years. All participants have a normal vision and none of them wore eyeglasses during the experiments.

### 3.3. Apparatus

**Figure 2:**
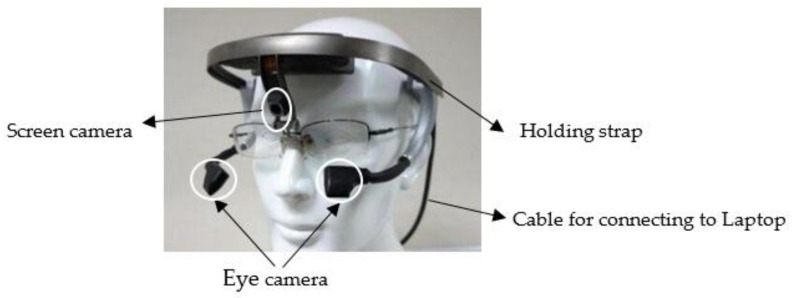
Eye Tracker device.

**Figure 3:**
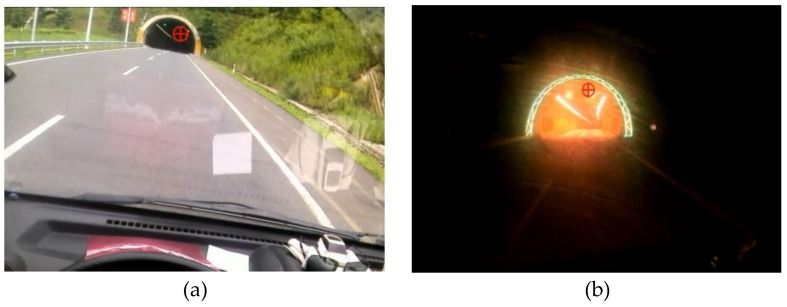
Road scene the driver sees (**a**) in the daytime; (**b**) in the nighttime.

The eye movements of the participants were recorded using a Dikablis Ergoneers Professional eye tracker (Ergoneers GmbH, Geretsried, Germany) at a sampling rate of 60 Hz (Figure 2). This device is a binocular eye tracker that can record left and right eye movements simultaneously. The head-mounted eye tracker device was equipped with a screen camera having a resolution of 1920 × 1080 pixels and two eye cameras having a resolution of 648 × 488 pixels. The pupil tracking accuracy was of 0.05° visual angle and the glance direction accuracy was of 0.1°–0.3° visual angle. The eye tracking data was recorded using a portable tablet and analyzed using the D-Lab software version 3.0 (Ergoneers GmbH, Geretsried, Germany) (it allowed for post-processed of the data, for instance, in case of sufficient pupil recognition) [71,72,73,74]. Figure 3 shows a typical driver’s view during daytime and nighttime captured by the screen camera. 

**Figure 4:**
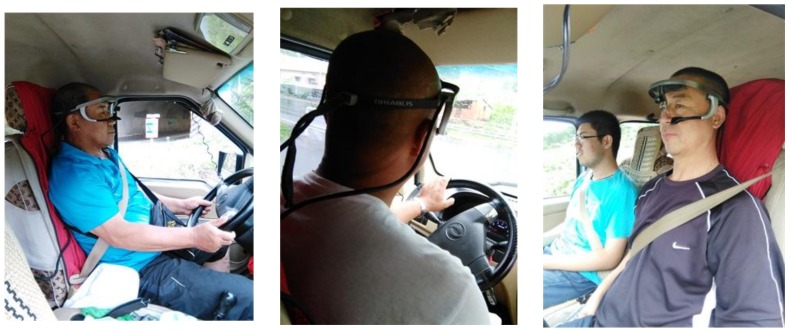
Participants wearing the eye-tracker device.

The vehicle is a front-wheel-drive, standard saloon car with manual transmission (Wuling Rongguang 6450B, SGMW, Guangxi, China). The vehicle weighs 1.2 tons, has an engine displacement of 1.2 L, a maximum torque of 112 Nm, and the engine produces a maximum power of 61 KW (83 Ps). Figure 4 shows a participant wearing the eye-tracker device.

### 3.4. Experimental Procedure

**Figure 5:**
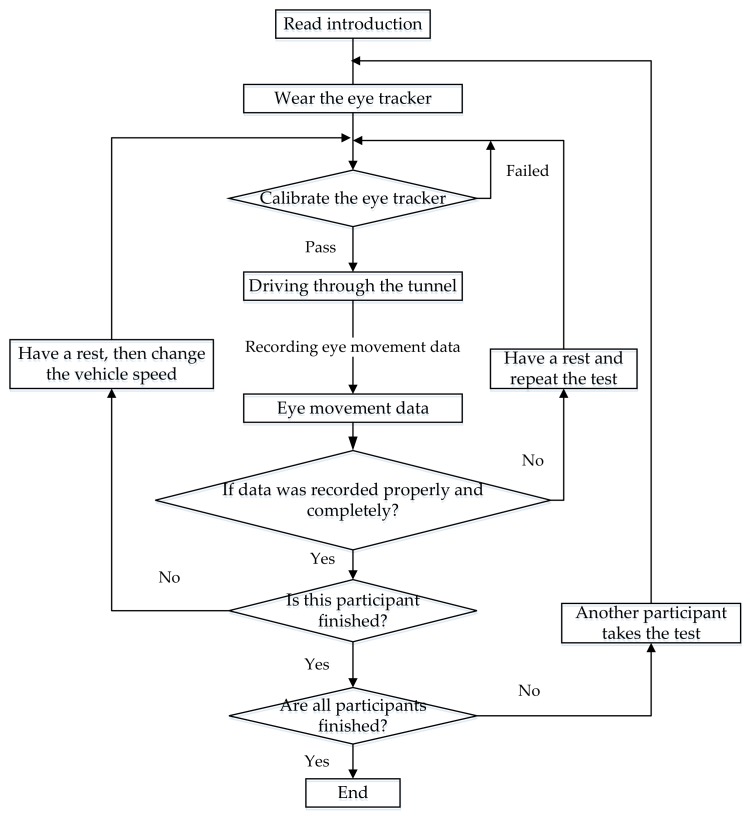
Flow Chart of the Experimental Procedure.

The tests were conducted at the end of July 2016. Figure 5 shows the flow chart of the experimental procedure followed for the daytime experiments. Before the experimental tests, each participant was briefed on the objective of the experiments, the experimental procedure and on the use of the eye tracker device. The experiments were conducted during daytime and nighttime. In the daytime experiments, each participant traversed from 200 m before the tunnel entrance to 200 m inside the tunnel [75,76] with predetermined constant speeds (40, 50, 60, 70 and 80 km/h). In the nighttime experiments, the traversed length was the same as that of the daytime experiments (Figure 5), however, only three speeds (40, 60 and 80 km/h) were used due to time limitations. 

**Figure 6:**
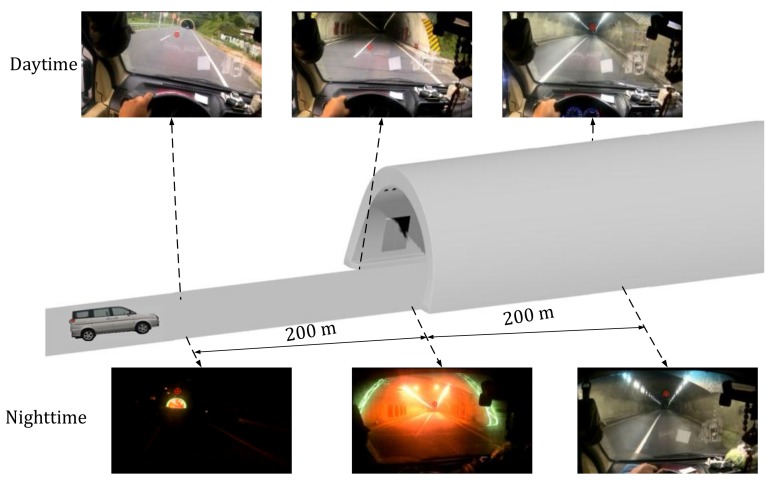
Drivers’ view at three different locations during the daytime and nighttime experiments.

Figure 6 depicts a sample of the driver’s view at three different locations during the daytime and nighttime experiments.

After a participant completed the experimental test, the technical personnel would check if the eye movement data was properly recorded. If this was not the case, the experiment was repeated with the same participant after a short break. It is pointed out that all experiments were completed on one sunny day. During the test, when overtaking occurred, the experimental technical personnel would make a mark on recording data for subsequent processing. In addition, the conditions for the road, weather, and the luminance in the tunnel were the same for all experiments. The only difference was the vehicle speed.

## 4. Results and Discussion

As mentioned earlier, the importance of visual perception in driving is often recognized as it is the input channel for sensory information [77]. Thus, it is important to investigate the drivers’ eye movement characteristic during driving. Three eye movement measures (pupil area, fixation duration and the number of fixations), which are considered to be basic eye movement parameters [78,79], were investigated in this work.

### 4.1. Data Processing

**Figure 7:**
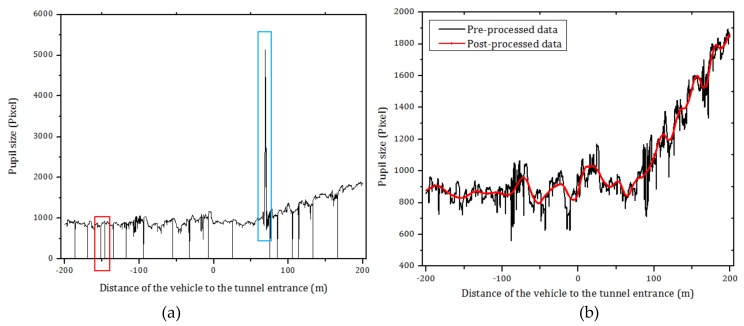
Sample of pre-processed and post-processed data for pupil size. (**a**) Original data of participant-A recorded by eye tracker device during daytime; (**b**) Post-processed and pre-processed data for pupil size of participant-A during daytime.

Before performing the statistical analysis, the original data (Figure 7a) acquired by the eye tracking device was post-processed to eliminate problems that may have occurred when using the device in realistic driving environments [80]. For instance, abnormally small pupillary in a short time (marked by a red rectangle in Figure 7a) may have been caused by eye blink, corneal reflection abnormality due to glare, changing illumination conditions, or other factors. In addition, the eye tracker may misidentify eyelashes into pupil holes when driver’s eyelashes are tight, and the color is similar to that of the pupil, and also would misidentify iris into pupil hole under changing illumination conditions, when the color of the iris is similar to that of the pupil, which would arise an abnormally large pupillary in a short time (marked by a blue rectangle in Figure 7a) [59]. Thus, we eliminated blinks and unlikely pupil sizes from the data before the statistical analysis (black line in Figure 7b). As observed in Figure 7, the pre-processed data for pupil diameter is noisy, which may be due to iris’ tremors, driver’s mental activity, among other factors [81]. To smooth this noisy data, a MATLAB wavelet transform was used in this paper. An example of the original and post-processed data are shown in Figure 7b. It should be noted that the significant individual difference in the eye movement parameters existed among individuals [82]. Besides, it is pointed out that all experimental figures after Figure 7 used post-processed data only. Moreover, the IBM SPSS Statistics 20.0 software was used in this paper to find the relationships between eye movement parameters, vehicle speed and the distance to the tunnel entrance.

### 4.2. Pupil Size

The human pupil size can regulate the amount of light that enters the eye [83]. The pupil size, which is the most popular eye-movement index for mapping mental workloads [84,85,86,87], not only reacts to luminance changes but also reflects cognitive processing. Moreover, pupil size is negatively correlated with the light conditions but is positively correlated with problem difficulty, that is, greater pupillary dilations are observed when the luminance is decreased or the difficulty of the problem is increased [88,89,90,91]. However, the changes in lighting are the main influencing factor [92].

**Figure 8:**
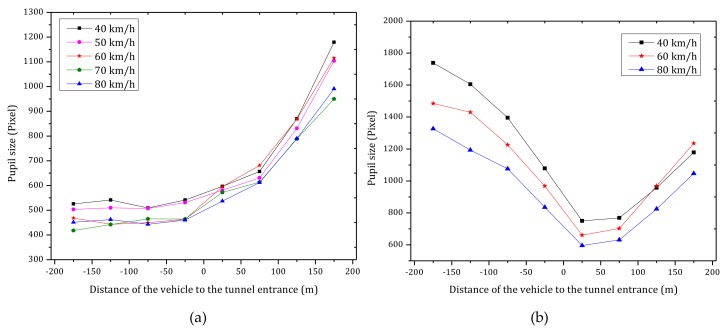
Average pupil size of the six participants under different vehicle speeds when driving into the tunnel entrance in (**a**) daytime; (**b**) nighttime.

Figure 8 shows the average pupil size of the six participants under different vehicle speeds when driving into the tunnel entrance in daytime and nighttime conditions. As observed in Figure 8a, in daytime, the pupil size remains pretty much constant before entering the tunnel entrance. However, the pupil size increases significantly after entering the tunnel entrance likely due to the sudden decrease of luminance at the tunnel entrance. As shown in Figure 8b, in nighttime, the pupil size decreases gradually before entering the tunnel entrance, likely because luminance increases as the vehicle approach the tunnel entrance. After entering the tunnel, the pupil size increases gradually, likely due to the luminance inside the tunnel is decreased. 

**Figure 9:**
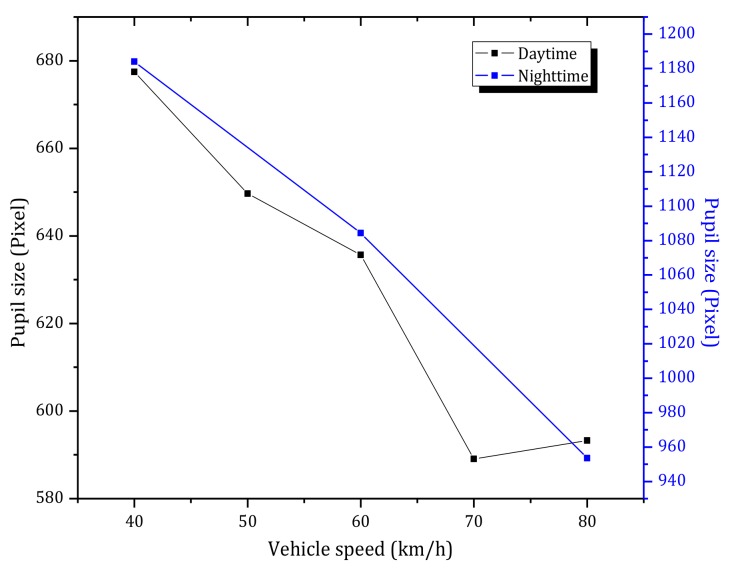
Average pupil size versus vehicle speed during daytime and nighttime.

Table 2 shows the Pearson coefficient between eye movement measures. Besides, it is pointed out the obtained *P*-values in the table are all corrected by means of Benjamini-Hochberg method [93]. As can be inferred from this table, mean pupil sizes in daytime and nighttime have negative correlations with vehicle speed. The latter means that pupil size is reduced, and hence drivers’ dynamic visual, with an increase of vehicle speed (see Figure 9). The reduction in pupil size may aim to improve dynamic visual acuity and to increase the ability to identify targets. 

Figure 8 shows that pupil size varies significantly when changing from bright to dark conditions (tunnel entrance in daytime, Figure 6) or when changing from dark to bright conditions (tunnel entrance in nighttime, Figure 6). In contrast, changes related to the vehicle speed have a smaller magnitude (see Figure 9).

### 4.3. Fixations

Fixation refers to maintaining of the visual gaze on a single location, which is also called fixation point [64]. This fixation point (e.g., red circle in Figure 3) is assumed to be the focus of attention. Fixation duration and the number of fixations are often used to describe drivers’ fixation behaviors [94,95].

#### 4.3.1. Fixation Duration

Fixation duration (ms) refers to the total amount of time for which a driver fixates on a specific area [94]. Overall, fixation duration is thought to be an indicator of a driver’s difficulty in extracting information. This means that a longer fixation duration on a specific area indicates drivers experience difficulty extracting or interpreting information from that area [96,97,98,99] and increases mental workload [100,101,102,103]. 

**Figure 10:**
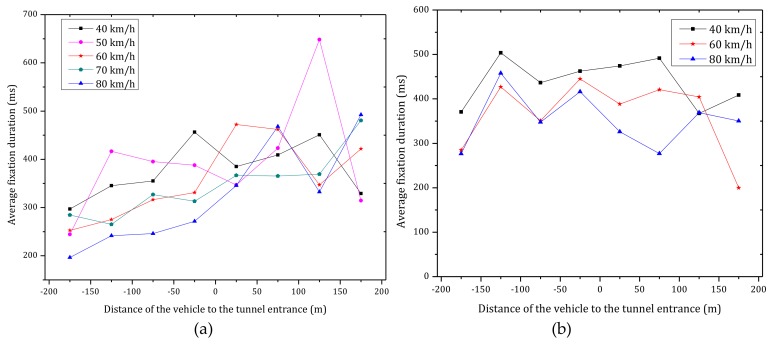
Average fixation duration versus distance of the vehicle to tunnel entrance for the six participants under various vehicle speeds during (**a**) daytime; (**b**) nighttime.

**Figure 11:**
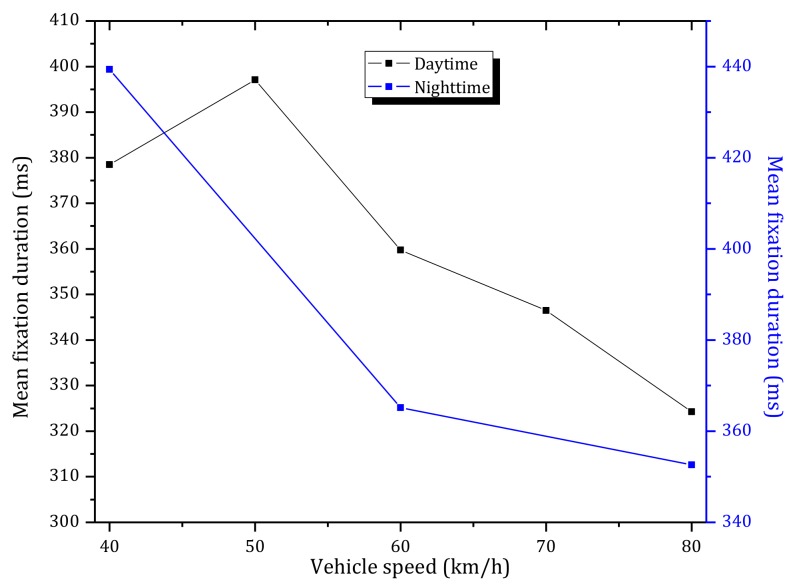
Mean fixation duration versus vehicle speed during daytime and nighttime.

Figure 10 shows the average fixation duration of six participants under different vehicle speeds at the tunnel entrance in daytime and nighttime. As observed in Figure 10a and Table 2, the average fixation duration consistently increased when entering the tunnel in daytime. The correlation coefficient between the average fixation duration and the distance to the tunnel entrance was found to be 0.943 (Pearson, *p* < 0.01). The latter results indicate that drivers are more focused inside the tunnel than outside of it. Furthermore, the average fixation duration has a negative correlation with the vehicle speed, which means that the average fixation duration decreased with an increase of the vehicle speed (black line in Figure 11). 

As shown in Figure 10b, in nighttime, there is no strong association between the average fixation duration and the distance of the vehicle to the tunnel entrance (*p* > 0.05). Furthermore, as shown in Figure 10b and Figure 11 (blue line), the average fixation duration is decreased with an increase in vehicle speed, however the statistical properties are not significant (Pearson, *p* > 0.05).

#### 4.3.2. Number of Fixations

The number of fixations means the total number of fixation points on an interest and area [104]. In driving, the number of fixations in a particular area reflects the importance of this area, which means, a larger number of fixation indicates more concern about this area [78]. In a visual search task, the number of fixations related to the amount of driving-related visual information processing, but it is unrelated to the depth of the interest [105].

**Figure 12:**
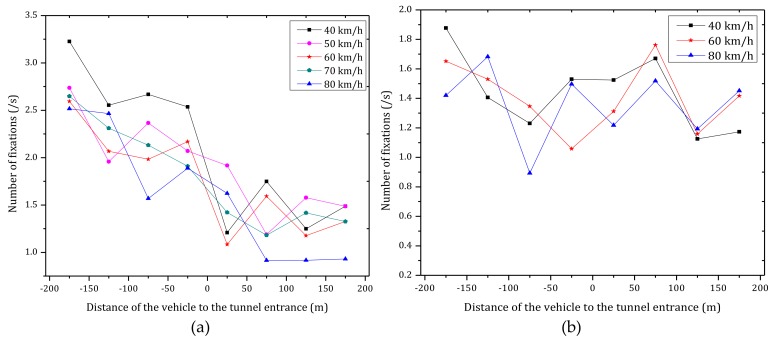
Average number of fixations per second of six participants under different vehicle speeds while approaching the tunnel entrance in (**a**) daytime; (**b**) nighttime.

Figure 12 shows the mean number of fixations per second of six participants under different vehicle speeds while approaching the tunnel entrance in daytime and nighttime. 

**Figure 13:**
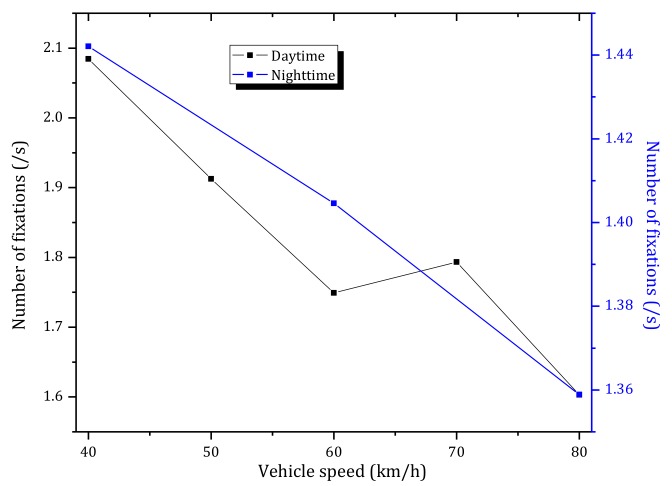
Number of fixations versus vehicle speed during daytime and nighttime.

As observed in Figure 12a, in daytime, the average number of fixations decreases significantly before tunnel entrance and then remains nearly constant after entering the tunnel. These results indicate that the drivers were more concerned about the transition zone when approaching the tunnel entrance. Furthermore, the number of fixations when approaching the tunnel entrance is significantly higher than inside the tunnel. In addition, the average number of fixations has a significant negative correlation with the distance to the tunnel (correlation coefficient −0.946, statistically significant at *p* < 0.01)). According to Figure 12a and Figure 13 (black line), the average number of fixations has a negative correlation with the vehicle speed, where the average number of fixations decreased with an increase of the vehicle speed. 

During nighttime, as can be observed in Figure 12b, there is not a strong correlation between the average number of fixations with the distance to the tunnel entrance. According to Figure 13 (blue line), the number of fixations decreased with an increase of the vehicle speed, but the statistical properties are not significant (Pearson, *p* > 0.05).

By comparing Figure 12a,b, it can be observed that the number of fixations in daytime is higher than nighttime, which is due to the outside environment in the daytime is more complex (luminance of sky, pavement and nearby buildings many have effects) than in the nighttime.

## 5. Conclusions

This paper is a pilot study (the sample size was small due to a combination of safety considerations, and limited time and funds) to examine data of drivers’ eye movements extracted from experiments performed in an actual highway tunnel to evaluate the effects of vehicle speed on drivers’ eye movement characteristics (pupil size, average fixation duration time and average number of fixation) at a highway tunnel entrance, and explored the variation characteristics of drivers’ eye movement when entering the tunnel entrance. The findings in this study showed that drivers’ pupil size changed slightly when approaching the tunnel entrance in the daytime, but had a sudden increase after entering the tunnel. Contrary to daytime, drivers’ pupil size decreased before entering the tunnel entrance and then increased gradually after entering the tunnel in nighttime. This indicates that the sudden decrease of the visual environment was reflected in an increase of drivers’ pupil size. Furthermore, the results showed that vehicle speed had a negative correlation with the pupil size in daytime and nighttime. This implies that pupil size of drivers is reduced to improve dynamic visual acuity, which in turn would increase the ability of drivers to identify targets. In addition, the average fixation duration in daytime consistently increased when entering the tunnel, but decreased with an increase of vehicle speed. However, the average fixation duration in nighttime didn’t have a strong association with the distance to the tunnel but had a negative correlation with vehicle speed. In addition, the number of fixations per second in daytime has a clear decreasing trend when entering a tunnel, which may indicate that drivers pay more attention to the external environment of the tunnel entrance. However, it was not found a strong association between the average number of fixations in nighttime with the distance to the tunnel entrance. Furthermore, the mean number of fixations in daytime and nighttime decreased with an increase of the vehicle speed. 

Thus, the lighting luminaries should be installed at the tunnel entrance in order to provide the appropriate luminance, which in turn would reduce the luminance difference problem as well as reduce the physical visual load of drivers. Besides, the luminance of the luminaries should be changeable, which can provide different luminance for tunnel lighting under the different weather. Additionally, it is recommended to install effective speed reduction markings, and that drivers comply with traffic signal warnings. In summary, this work discussed the effects of vehicle speed on drivers’ eye movement features based on experiments performed in an actual highway tunnel. This study provides an experimental basis for further research on drivers’ eye movement characteristics, which is of significance to improve driving safety and avoid traffic accidents in highway tunnels. 

Although the results presented in the paper are certainly promising, the limitations of the experiment should be addressed. The main limit of the tests includes the small sample size and doesn’t consider counterbalancing techniques to avoid the practice effect. Thus, the future studies should enlarge the sample of case studies for proposing a convincing result. Besides, some counterbalancing techniques should be considered in the future studies. And future studies could be carried out under different weather conditions to assess the differences between novice and experienced drivers, between male and female drivers, among other parameters.

## Figures and Tables

**Table 1:** Basic information of participants.

Participant	Gender	Age	Type of Driver License	Driving Experience (Years)
A	male	36	A	14
B	male	37	A	12
C	male	39	A	16
D	male	58	A	32
E	male	32	A	13
F	male	35	A	12

**Table 2:** Pearson coefficient and *P* value showing a correlation between vehicle speed, distance to the tunnel entrance and the eye movement parameters.

Measure	Speed	Distance	Daytime PZ	Nighttime PZ	Daytime AFD	Nighttime AFD	Daytime MNF	Nighttime MNF
Speed	1	/	−0.960 *p* = 0.054	−0.997 *p* = 0.123	−0.893 *p* = 0.111	−0.957 *p* = 0.419	−0.946 *p* = 0.062	−0.990 *p* = 0.177
Distance	/	1	0.878 * *p* = 0.027	−0.649 *p* = 0.170	0.943 ** *p* = 0.000	−0.344 *p* = 0.736	−0.946 ** *p* = 0.000	−0.380 *p* = 0.562
Daytime PZ	−0.960 *p* = 0.054	0.878 * *p* = 0.027	1	−0.234 *p* = 0.736	0.691 *p* = 0.131	−0.465 *p* = 0.419	−0.736 *p* = 0.111	−0.282 *p* = 0.672
Nighttime PZ	−0.997 *p* = 0.123	−0.649 *p* = 0.170	−0.234 *p* = 0.736	1	−0.807 *p* = 0.062	−0.214 *p* = 0.736	0.784 *p* = 0.071	0.202 *p* = 0.736
Daytime AFD	−0.893 *p* = 0.111	0.943 ** *p* = 0.000	0.691 *p* = 0.131	−0.807 *p* = 0.062	1	0.050 *p* = 0.964	−0.978 ** *p* = 0.000	−0.346 *p* = 0.591
Nighttime AFD	−0.957 *p* = 0.419	−0.344 *p* = 0.736	−0.465 *p* = 0.419	−0.214 *p* = 0.736	0.050 *p* = 0.964	1	0.033 *p* = 0.964	−0.019 *p* = 0.964
Daytime MNF	−0.946 *p* = 0.062	−0.946 ** *p* = 0.000	−0.736 *p* = 0.111	0.784 *p* = 0.071	−0.978 ** *p* = 0.000	0.033 *p* = 0.964	1	0.336 *p* = 0.591
Nighttime MNF	−0.990 *p* = 0.177	−0.380 *p* = 0.562	−0.282 *p* = 0.672	0.202 *p* = 0.736	−0.346 *p* = 0.591	−0.019 *p* = 0.964	0.336 *p* = 0.591	1

PZ = pupil size; AFD = Average fixation duration; MNF = Mean number of fixations per second; ** Significant at the 0.01 level; * Significant at the 0.05 level.
